# Extraintestinal Manifestation of *Yersinia pseudotuberculosis* Bacteremia as Acute Hepatitis: Case Report and Review of the Literature

**DOI:** 10.3390/pathogens10101255

**Published:** 2021-09-28

**Authors:** Yun Jeong Lee, Jooyun Kim, Ji Hoon Jeon, Hyeri Seok, Won Sun Choi, Eun-Ah Chang, Hyung Joon Yim, Dae Won Park

**Affiliations:** 1 Department of Internal Medicine, Korea University Ansan Hospital, Korea University College of Medicine, Ansan-si 15355, Korea; leey217@gmail.com; 2 Division of Infectious Diseases, Department of Internal Medicine, Korea University Ansan Hospital, Korea University College of Medicine, Ansan-si 15355, Korea; jooyuna777@gmail.com (J.K.); jjhinlove@naver.com (J.H.J.); cmcws@korea.ac.kr (W.S.C.); pugae1@korea.ac.kr (D.W.P.); 3 Department of Laboratory Medicine, Korea University Ansan Hospital, Korea University College of Medicine, Ansan-si 15355, Korea; crlmember@naver.com; 4 Division of Gastroenterology and Hepatology, Department of Internal Medicine, Korea University Ansan Hospital, Korea University College of Medicine, Ansan-si 15355, Korea; gudwns21@korea.ac.kr

**Keywords:** *Yersinia pseudotuberculosis*, hepatitis, bacteremia, Yersinia infections, Yersinia

## Abstract

*Yersinia pseudotuberculosis* is a causative agent of foodborne zoonosis that usually causes self-limiting pseudoappendicitis. *Y. pseudotuberculosis* infection also causes systemic spread or extraintestinal manifestations in patients with predisposing conditions. Here, we present a case of acute hepatitis with *Y. pseudotuberculosis* bacteremia in a 30-year-old man. He was previously healthy without significant medical history other than obesity and current smoking. At the time of admission, he presented with high fever accompanied by chills, jaundice, abdominal pain, and watery diarrhea. Laboratory studies revealed leukocytosis and elevated liver function parameters. A stool culture showed no causative pathogens. Empiric antibiotic therapy with ceftriaxone and metronidazole was administered. *Y. pseudotuberculosis* was later isolated from the initial blood culture performed on the day of admission using MALDI-TOF mass spectrometry. Antibiotic treatment was continued based on the susceptibility testing results from MALDI-TOF MS and VITEk^®^2, as well as clinical and laboratory improvements. The patient was discharged on the tenth day of admission and remained healthy with no recurrence during the 12-month follow-up. Here, we review the literature on the systemic infection caused by *Y. pseudotuberculosis*, including extraintestinal manifestations. This case highlights that *Y. pseudotuberculosis* may be considered a differential causative organism in patients with acute colitis and hepatitis.

## 1. Introduction

*Y. pseudotuberculosis* infection has been primarily reported in temperate regions, such as North America, Europe, and Japan, rather than in tropical or subtropical regions [1]. In healthy patients, *Y. pseudotuberculosis* typically causes self-limiting terminal ileitis, often accompanied by mesenteric lymphadenitis. A severe systemic inflammatory variant of the disease, called Far East scarlet-like fever (FESLF), has occurred in Russia and Japan [2]. *Y. pseudotuberculosis*-derived mitogen (YPM), a superantigenic toxin produced by Far Eastern strains, has been suggested to disrupt the host immune system due to reasons related to the pathogenesis of its systemic manifestation [3]. The major symptoms of FESLF include hyperemic tongue, pharyngotonsillitis, rash with desquamation, lymphadenopathy, arthralgia, and generalized symptoms such as fever and chills.

Bacteremia rarely occurs in *Y. pseudotuberculosis* infections [4], and such cases have usually been reported in patients with underlying illness including liver cirrhosis, diabetes, hemochromatosis, thalassemia, malignancy, and immune suppression [5]. Although hepatic involvement is uncommon in most cases of *Y. pseudotuberculosis* infections, acute hepatitis has been reported in half of FESLF patients [2]. Here, we describe a rare case of acute hepatitis with *Y. pseudotuberculosis* bacteremia other than FESLF in a patient with no underlying medical conditions other than obesity and current smoking.

## 2. Case Presentation

A 30-year-old man without significant medical history presented to the emergency room with fever lasting 3 days. He complained of chills, abdominal pain, watery diarrhea, nausea, and dyspepsia. He denied vomiting, constipation, general weakness, unintentional weight loss, headache, cough, sputum production, shortness of breath, chest pain, increased urinary frequency, or dysuria. He was obese, with a height of 173.4 cm, a weight of 103.4 kg, and a BMI of 34.0 kg/m^2^. He ascribed his symptoms to his 3-days prior drinking of a cup of Americano that had been made at a franchise café and left at room temperature for several days. There was no other unusual food ingestion, including raw or undercooked meals. Recent travel or contact with ill individuals was also denied. There was no history of alcohol or drug use. He was a current smoker with 10 pack-years, and his family history was unremarkable. He had no known food or drug allergies.

On initial physical examination, he appeared acutely ill, with a temperature of 38.4 °C, a blood pressure of 124/57 mmHg, a heart rate of 75/min, a respiratory rate of 16/min, and an oxygen saturation of 100% in room air. He was alert and oriented to time, person, and place. Head, eyes, ears, nose, and throat examination was remarkable for icteric sclera, but there was no obvious tonsil enlargement, sinus tenderness, neck rigidity, or enlarged lymph nodes. Lungs were clear to auscultation. Cardiac auscultation revealed a regular rate and rhythm without murmurs, rubs, or gallops. There was localized tenderness in the lower abdomen, but he had no guarding, rebound tenderness, masses, or distension. The bowel sounds were diminished. The remainder of the physical examination was unremarkable.

**Figure 1:**
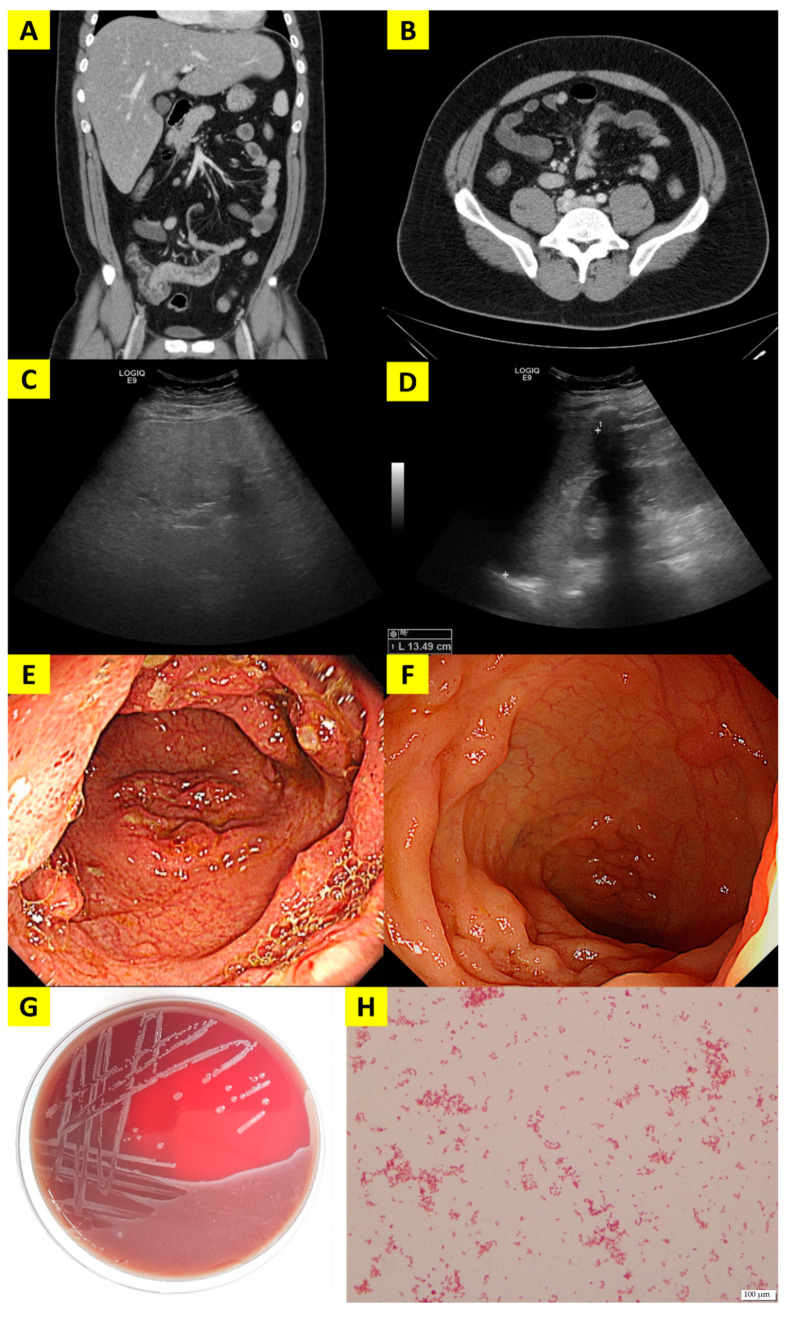
(**A**) Contrast-enhanced abdominal CT scan showing marked wall thickening and mucosal hyper-enhancement in the terminal ileum and minimal intussusception at the ileocecal valve, along with (**B**) multiple enlarged mesenteric lymph nodes. (**C**) Ultrasound showing markedly increased liver echogenicity (**D**) and mildly enlarged spleen at 13 cm. (**E**) Total colonofiberscopy showing hyperemic nodular edematous mucosa on the terminal ileum and hemorrhagic spots on the rest of the colon, (**F**) which improved after two months in follow-up colonoscopy. (**G**) Colonies of *Yersinia pseudotuberculosis* grown on blood agar plate. (**H**) Short, plump, Gram-negative bacilli visualized under light microscopy.

Initial laboratory investigations indicated an elevated white blood cell count (12,590 cells/mm^3^) with 80.3% neutrophils, a C-reactive protein level of 9.340 mg/dL, an elevated aspartate aminotransferase level of 392 IU/L, an alanine aminotransferase level of 136 IU/L, a γ-glutamyl transferase level of 487 IU/L, and a slightly prolonged prothrombin time of 12.7 s. Chest radiography findings were unremarkable. A contrast-enhanced abdominal CT scan showed marked wall thickening and mucosal hyper-enhancement in the terminal ileum (Figure 1A), as well as minimal intussusception at the ileocecal valve with multiple enlarged mesenteric lymph nodes along the celiac axis and aortocaval, left paraaortic, and ileocecal areas (Figure 1B).

The initial impressions upon admission were acute colitis and hepatitis A. The patient was started on 2 g of intravenous (IV) ceftriaxone q24h plus 500 mg of IV metronidazole q8h as empiric antibiotics to cover for acute colitis.

The following tests were performed to determine the cause: stool culture, repeated hepatitis A virus IgM, anti-hepatitis B core IgM, cytomegalovirus, Epstein–Barr virus, human immunodeficiency virus, and fluorescent antinuclear antibody. All of them turned out to be negative. The ultrasound showed markedly increased liver echogenicity (Figure 1C) and a mildly enlarged spleen at 13 cm (Figure 1D). A total colonofiberscopy showed hyperemic nodular edematous mucosa on the terminal ileum and hemorrhagic spots on the rest of the colon, with biopsy findings of chronic active inflammation with ulcers (Figure 1E).

The blood culture obtained on the day patient visited the emergency room was detected as positive on the BACT/ALERT^®^ VIRTUO^®^ (bioMérieux, Lyon, France) system. For identification and antibacterial susceptibility testing, we applied the matrix-assisted laser desorption/ionization time-of-flight mass spectrometry (MALDI-TOF MS) (VITEK-MS, bioMérieux, Lyon, France) and VITEK^®^2 with the GN and AST 224 cards (bioMérieux, Lyon, France). The final report confirmed *Y. pseudotuberculosis*, which was susceptible to all the reference antimicrobial agents, including third-generation cephalosporins (Table 1). The blood agar plate inoculated with a small part of the positive blood culture showed gray translucent colonies. (Figure 1G) Gram-stained smears showed pleomorphic, Gram-negative plump rods as single cells (Figure 1H).

The fever started to decline on the day following admission and completely resolved on the eighth day of antimicrobial therapy. Diarrhea gradually decreased to three times per day and further subsided on the eighth day. The subsequent blood culture showed no growth of *Y. pseudotuberculosis*. On day 9, all symptoms resolved. The laboratory parameters, including white blood cell counts, CRP levels, and liver function, had improved to lie between the reference ranges on the 10th day of admission. After 10 days of antibiotic therapy and conservative management, the patient was uneventfully discharged. There was no recurrence during the follow-up for 12 months.

## 3. Discussion

*Yersinia pseudotuberculosis* (a motile, non-spore-forming, facultative anaerobic, Gram-negative coccobacillus) has a broad range of animal reservoirs, including both domesticated and wild mammals and birds [6]. Following the ingestion of fecally contaminated food or water, the microorganism mainly affects the right colon, ileum, or appendix by binding to the intestinal wall and initiating systemic invasion through Peyer’s patches of the mesenteric lymph nodes, spleen, and liver. The most common symptoms are fever, diarrhea, and abdominal pain, predominantly in the right lower quadrant and thus mimicking appendicitis. The endoscopic finding of *Y. pseudotuberculosis* enteritis reveals linear aphthous mucosal ulceration in the terminal ileum. Microscopic findings show localized granuloma formation, with central necrosis accompanied by inflammatory nodules centered on Peyer’s patches and mesenteric lymphadenopathy [7].

Outbreaks of serious systemic infection, identified as FESLF and caused by a severe form of *Y. pseudotuberculosis* infection, have occurred [8]. The lipopolysaccharide core of *Y. pseudotuberculosis* mediates the expression of CD209 receptors on antigen-presenting cells, which explains bacterial dissemination to the mesenteric lymph nodes, spleen, and liver [9]. Few cases of *Y. pseudotuberculosis* infection have been reported in Korea, and they have mostly shown gastrointestinal manifestation [10,11]. Because most *Y. pseudotuberculosis* infections are mild and self-limiting and the isolation of the pathogen is difficult, the spread of the bacteria may have been underestimated; furthermore, extraintestinal manifestations of *Y. pseudotuberculosis* infection are rarely reported.

The patient was young and had no significant medical history without previously reported risk factors for *Y. pseudotuberculosis* bacteremia such as liver cirrhosis, diabetes, hemochromatosis, thalassemia, or malignancy. Here, we assumed that obesity and smoking were the only conditions that might contribute to the generalized form of *Y. pseudotuberculosis* infection. Previous studies have shown that obesity is associated with an increased susceptibility to infections and their severity [12]. A possible pathophysiology is known, in that obesity violates the well-balanced crosstalk between adipocytes and immune cells, with the subsequent dysregulation of immune surveillance system; for example, the altered secretion of inflammatory mediators decreases cell-mediated immune responses. Additionally, cigarette smoking is known to disrupt the protective function of small intestine and colon by inducing cell apoptosis and reducing mucosal blood flow [13].

The route of transmission in this patient remained unclear. He insisted that he kept a regular life style in urban areas and a routine diet in similar places. He denied any unreliable access to drinking water except for the cup of Americano left for several days. Additionally, he reported no contact with wild animals or pets. Considering the fact that the incubation period of *Y. pseudotuberculosis* infection is usually 4–10 days after ingestion [14], we could not determine a possible source of contamination within a month before the onset of the patient’s symptoms through interview.

In this case, *Y. pseudotuberculosis* infection was diagnosed with a blood culture performed on the day of admission before starting antibiotics. Additionally, the application of MALDI-TOF MS on the early growth from the culture made the rapid and accurate identification of *Y. pseudotuberculosis* feasible. Although *Y. pseudotuberculosis* grows on many types of routine agar incubated at 35 °C in ambient air, other enteric bacteria in the setting can overgrow it. The low reported incidence of human infections of *Y. pseudotuberculosis* is possibly due to the fact that systematic cefsulodin–irgasan–novobiocin agar, incubated at 25–28 °C (which can help selectively culture *Y. pseudotuberculosis*), is not systematically used in diarrheal patients [15]. Additionally, most gastrointestinal pathogen PCR panels determine the presence of *E. coli, Salmonella* spp., *Shigella* spp., *Vibrio* spp., *Campylobacter* spp., and *Y. enterocolitica*, so *Y. pseudotuberculosis* may hardly be found in routine diarrheal tests. Thus, the application of MALDI-TOF MS in identifying pathogens can help estimate the hidden prevalence of *Y. pseudotuberculosis* infections. According to a report in 2010 [12], a high proportion of *Y. pseudotuberculosis* cases have been diagnosed by blood culture, which suggests that underdiagnoses of less invasive *Y. pseudotuberculosis* infections may explain the rarity of its incidence. Furthermore, the fact that *Y. pseudotuberculosis* and its natural history are very unfamiliar to physicians makes the clinical diagnosis more challenging.

We reviewed the literature of *Y. pseudotuberculosis* bacteremia published between 2000 and 2021 (Table 2). All but one case were adults, the age distribution varied, and more cases were reported in men. In 4 of the 12 reported cases, the patients had no underlying diseases, and among the remaining reports, the most common underlying diseases were chronic hepatitis c viral infection, liver cirrhosis, colon cancer, solid organ transplantation, human immunodeficiency virus (HIV) infection, and other immunocompromised status that may be considered risk groups. Reported possible transmission routes were spring water, half-roasted barbecue, and iceberg lettuce. Fever with gastrointestinal symptoms were most common, three cases only presented extraintestinal symptoms, and two cases of septic arthritis and one case of vertebral osteomyelitis were diagnosed. Acute hepatitis as an extraintestinal manifestation has not been reported. If antimicrobial susceptibility tests were described, *Y. pseudotuberculosis* was not found to be multidrug-resistant and was sensitive to ampicillin, tetracycline, chloramphenicol, cephalosporin, fluoroquinolones, and aminoglycosides [13]. Cases generally showed excellent outcomes with ceftriaxone with or without ampicillin, ciprofloxacin, or gentamicin.

Although *Y. pseudotuberculosis* is not commonly included in the differentials for acute hepatitis, in the present case, the infection was manifested not only as acute enterocolitis but also as jaundice and showed elevated liver function parameters. This highlights that *Y. pseudotuberculosis* infection may appear as unusual manifestations other than enteritis associated with systemic involvement. In conclusion, *Y. pseudotuberculosis* hepatitis may be considered a differential diagnosis in patients with underlying diseases showing not only signs of acute onset hepatitis but also those of infectious colitis. Additionally, we expect that the development of diagnostic technologies such as the MALDI-TOF MS used here will broaden our understanding of the actual prevalence and natural history of such uncommon pathogens.

## Figures and Tables

**Table 1:** Identification and antibacterial susceptibility testing results of the purified colonies from blood culture using VITEK 2.

**Method Name**	VITEK^®^2	
Organism Name	*Yersinia Pseudotuberculosis*	
Antibiotic	Antibiotic Family	Interpretation
Amikacin	Aminoglycosides	S
Amoxicillin/Clavulanic Acid	Amino-penicillins and beta-lactam inhibitors	S
Ampicillin	Amino-penicillins	S
Aztreonam	Monobactam	S
Cefazolin	Cephalosporins 1	S
Cefepime	Cephalosporins 4	S
Cefotaxime	Cephalosporins 3	S
Cefoxitin	Cephamycins	S
Ceftazidime	Cephalosporins 3	S
Ciprofloxacin	Fluoroquinolones	S
Ertapenem	Penems	S
Gentamicin	Aminoglycosides	S
Imipenem	Penems	S
Tigecycline	Tetracyclines	S
Trimethoprim/Sulfamethoxazole	Trimethoprim/Sulfonamides	S

**Table 2:** Literature review of publications on *Yersinia pseudotuberculosis* bacteremia covering the years 2000–2021.

Author/Reference	Year Published/Location	Age/Gender	Underlying Diseases	Possible Transmission Route	Clinical Manifestations	Site of Infection	Antibiotic Treatment	Outcome
Hashimoto et al. [16]	2021/Japan	38 y/Male	Mood disorder	Half-roasted barbecue	Fever, vomiting, diarrhea	Gastroenteritis	Ceftriaxone and Azithromycin → Cefmetazole	Recovered
Kamura et al. [17]	2020/Japan	10 mo/Male	None	Spring water	Fever, diarrhea, and Kawasaki disease-like symptoms	Gastroenteritis	Ampicillin and Ceftriaxone → Meropenem and IVIG →Ampicillin	Recovered
Martyn et al. [18]	2020/United Kingdom	76 y/Male	None	Not explicable	Fever and arthritis	Septic arthritis at the knee joint	Ceftriaxone	Recovered
Harch et al. [19]	2019/Australia	73 y/Female	Colorectal cancer, stage IV breast cancer, vulvar cancer, and type 2 diabetes mellitus	Not explicable	Fever and diarrhea	Gastroenteritis	Ceftriaxone and Ciprofloxacin → Ciprofloxacin	Recovered
Renvoisé et al. [4]	2015/France	49 y/Male	Liver transplantation Liver cirrhosis due to chronic hepatitis C viral infection	Not explicable	Fever, diarrhea, and confusion	Spontaneous bacterial peritonitis	Amoxicillin/Clavulanate and Ciprofloxacin → Ceftriaxone and Ciprofloxacin → Ceftriaxone and Gentamicin	Recovered
Kaasch et al. [20]	2012/Germany	42 y/Male	Burkitt lymphoma	Not explicable	Fever	Hip joint	Ceftriaxone → Ciprofloxacin	Recovered
Mischnik et al. [21]	2012/Germany	73 y/Male	Hemophilia A, heart failure, valvular heart disease, peripheral artery occlusive disease, ischemic heart disease, chronic kidney disease, and chronic hepatitis C viral infection	Not explicable	Fever and arthralgia	Knee joint	Piperacillin/tazobactam and Clarithromycin	Deceased
Mashiba et al. [22]	2008/Japan	22 y/Female	None	Not explicable	Fever, fatigue, and exanthema	Primary bacteremia	Imipenem	
Vincent et al. [23]	2008/France	20 patients (17–83 years; median: 73 y)	Colorectal cancer, diabetes, liver cirrhosis, multiple myeloma, kidney transplantation, and chronic hepatitis C viral infection	Not explicable	Fever, diarrhea, and shock	Gastroenteritis	Not explicable	13 Recovered 7 Deceased
Paglia et al. [5]	2005/Italy	42 y/Female	HIV infection	Not explicable	Fever, confusion	Gastroenteritis	Ceftriaxone	Recovered
		54 y/Male	HIV infection	Not explicable	Fever, confusion	Gastroenteritis	Ceftriaxone	Recovered
Nuorti et al. [24]	2004/Finland	47 patients (2–77 years; median: 19 y)	None	Iceberg lettuce	Fever, abdominal pain, diarrhea	Gastroenteritis	Not explicable	46 Recovered 1 Deceased
Van Zonneveld et al. [25]	2002/Netherlands	54 y/Male	Kidney transplantation due to focal segmental glomerulosclerosis	Not explicable	Fever	Infectious spondylitis, L1, and splenic abscess	Cefuroxime → Ciprofloxacin	Recovered

## Data Availability

Data sharing not applicable.
